# Occipital Condyle Fracture with Accompanying Meningeal Spinal Cysts as a result of Cervical Spine Injury in 15-Year-Old Girl

**DOI:** 10.1155/2015/627502

**Published:** 2015-10-12

**Authors:** Łukasz Wiktor, Ryszard Tomaszewski

**Affiliations:** Department of Paediatric Orthopaedics, Silesian Medical University, Katowice, Poland

## Abstract

The occipital condyle fracture is rare injury of the craniocervical junction. Meningeal spinal cysts are rare tumors of the spinal cord. Depending on location, these lesions may be classified as extradural and subdural, but extradural spinal cysts are more common. We present the case of a 15-year-old girl who suffered from avulsion occipital condyle fracture treated with use of “halo-vest” system. We established that clinical effect after completed treatment is very good. Control MRI evaluation was performed 12 months after removal of “halo-vest” traction, and clinically silent extradural meningeal spinal cysts were detected at the ventral side of the spinal cord in the cervical segment of the spine. Due to clinically silent course of the disease, we decided to use the conservative treatment. The patient remains under control of our department.

## 1. Introduction 

The occipital condyle fracture is rare injury of the craniocervical junction, which is more commonly diagnosed in adult persons. Most commonly, these fractures are caused by traffic accident. The best imaging evaluation used for establishing diagnosis is CT-scan of C0-C1-C2 junction. Treatment method depends on morphology and stability of the fracture. The literature describes many accompanying injuries and complication of injuries of craniocervical junction; however, there is no description of accompanying extradural meningeal cyst. The meningeal cysts occur very rarely and they constitute only 1% of all tumors of the spine. Etiology of these lesions is not entirely known; however, in some cases, it is associated with past injury. Posttraumatic cysts may occur at any level of the spinal cord, but, most frequently, they are located in the central and inferior part of the thoracic segment of the spine. The best diagnostic tool for diagnosis of the extradural cysts of the spinal cord is MRI of the spine. Treatment of the meningeal cyst depends on its size, location, and accompanying neurological symptoms.

## 2. Case Report

**Figure 1:**
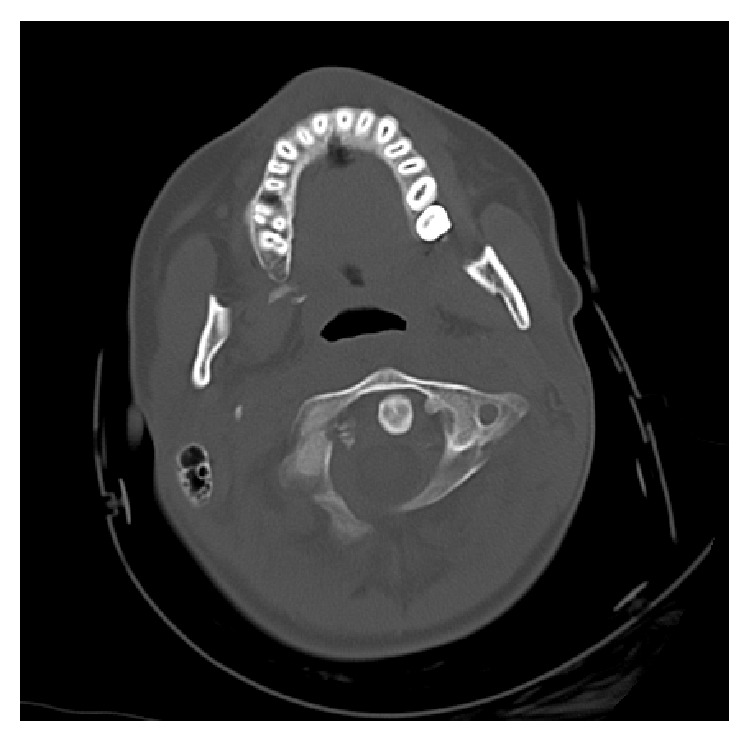
Axial CT reconstruction image shows avulsion fracture of the right occipital condyle (type III according to Anderson-Montesano classification).

**Figure 2:**
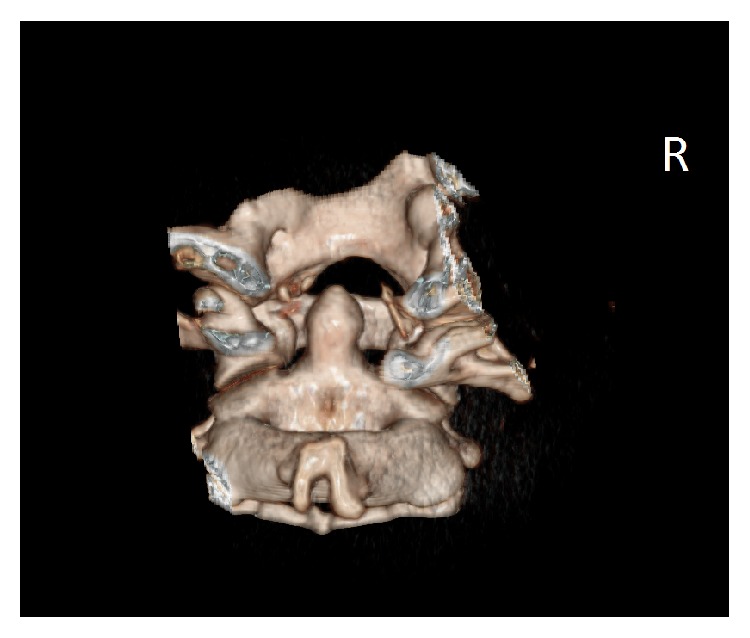
Three-dimensional CT reconstruction image shows avulsion fracture of the right occipital condyle (back view).

**Figure 3:**
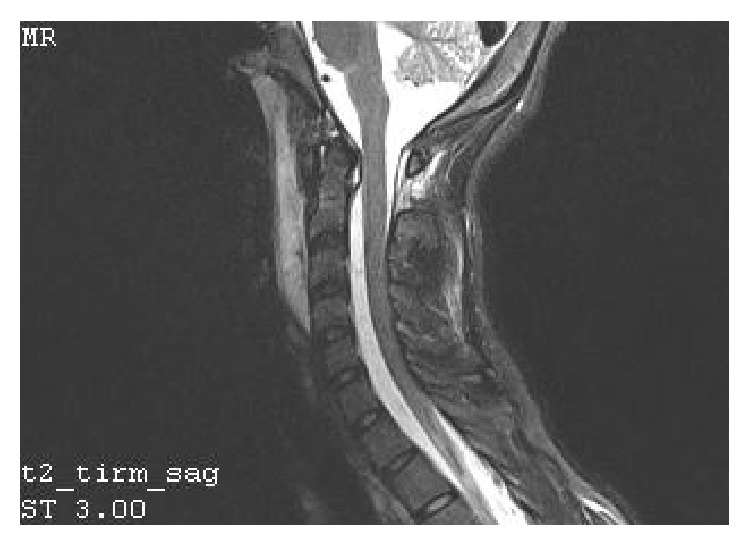
Sagittal MR reconstruction image shows spinal cord after trauma.

**Figure 4:**
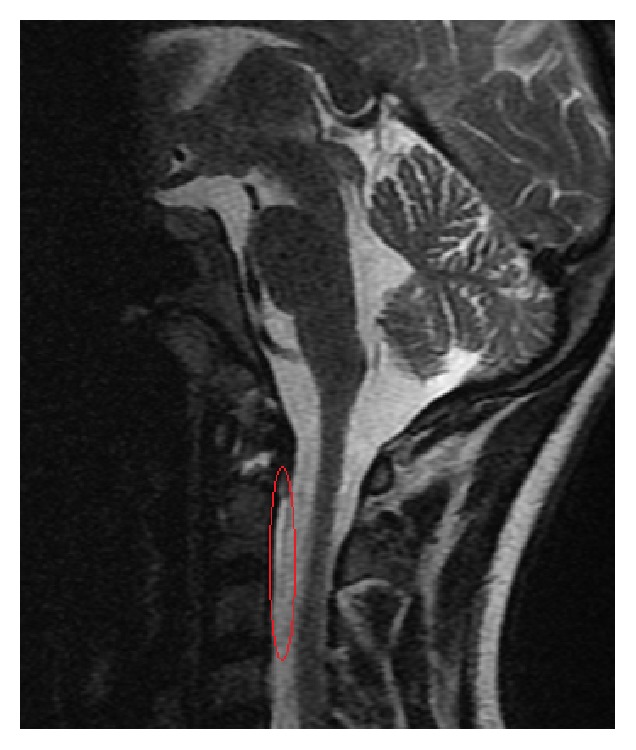
Sagittal MR reconstruction image shows narrow fluid compartment at the level of C2-C3 (marked), poor quality due to the stabilization of the “halo-vest.”

**Figure 5:**
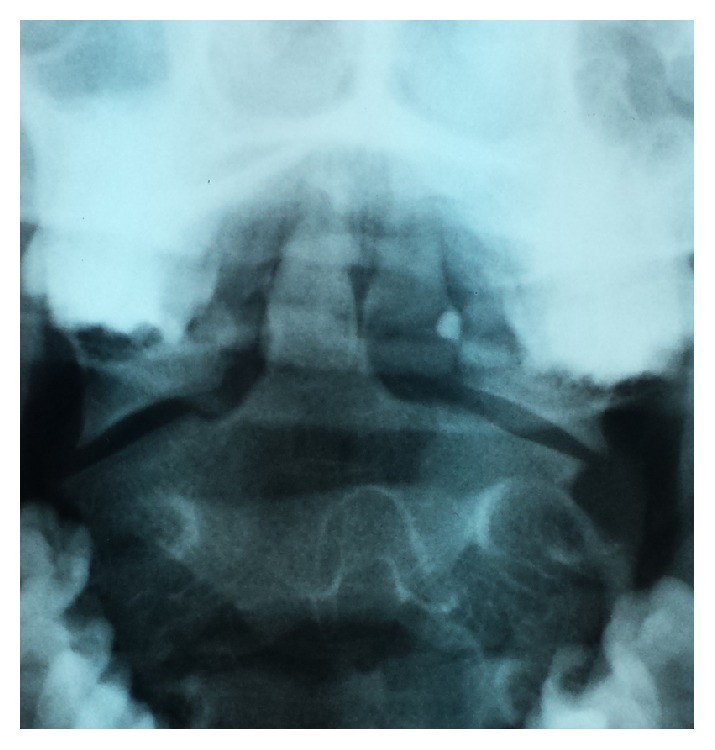
AP “open mouth view” after removing “halo-vest” system.

**Figure 6:**
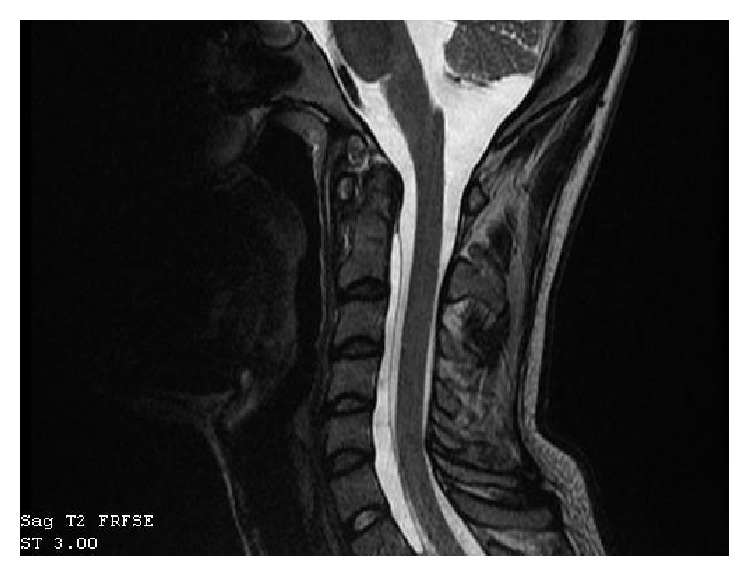
Sagittal MR reconstruction image shows an extradural meningeal spinal cyst.

A 15-year-old girl experienced injury of the head and the cervical segment of the spine as a result of being hit by a car. Indirect injury of the craniocervical junction resulted from the fall in bend-rotation mechanism. The patient was initially managed at the site of incident by the emergency medical service, and she was transported to the emergency department. At the moment of admission, the patient was conscious and confused and with GCS score of 13 points. Based on conducted imaging diagnostics (trauma scan CT before and after intravenous administration of the contrast medium), the following diagnosis was established: multifocal injury, pulmonary contusion with laceration, and the right occipital condyle fracture (Figures 1 and 2). General condition of the patient was stable, and decision was made to transfer the patient to the Center of Pediatric Traumatology. The diagnostics in our department was extended with MRI of the head and MRI of the cervical segment of the spine in order to evaluate the ligament system of C0-C1-C2 junction (Figure 3) and control evaluation with CT-scan of the head. Based on aforementioned evaluations, the following diagnosis was established: avulsion fracture of the right occipital condyle (type III according to Anderson-Montesano classification). Considering unstable nature of the fracture, the patient was qualified for treatment with use of an external fixation of “halo-vest” type. Paraprocedural and postprocedural course were not complicated. “Halo-vest” fixation was maintained over 13 weeks with performed control of local condition of the skin within the area of the pins and neurological condition of the patient focused on the inferior cranial nerves IX–XII. Within the 5th week of treatment, control X-ray of the cervical segment of the spine and MRI evaluation were performed, based on which maintained asymmetry was diagnosed in the medial atlantoaxial joint and signs of progressing adhesion at the level of the right alar ligament. In the vertebral canal at the level of C2-C3 at the front of the spinal cord, in extradural location, mainly at the left side, narrow fluid compartment was visualized (Figure 4). Treatment was not complicated. “Halo-vest” system was removed. X-ray of the cervical spine (AP + LATERAL + AP open mouth view, Figure 5) was performed as well as functional X-ray in anteflexion and retroflexion based on which no signs of instability were established at the level of C0-C1-C2. The patient received an additional treatment with the cervical collar over 4 weeks. After 20 weeks following injury, clinical evaluation was performed; the patient did not report any pain within the cervical spine. NDI (Neck Disability Index) was established with exclusion of point 8 (car driving) obtaining the result of 3/45 (6.7%) which allowed ruling out disability caused by pain of the cervical segment of the spine. 12 months after removal of “halo-vest” system, control MRI evaluation of the craniocervical junction revealed significant enlargement of previously described fluid cistern. Extradural meningeal spinal cyst was diagnosed, which was located between the levels of C2 and C7, measuring up to 6 mm thick in the largest dimension (Figure 6). Due to lack of clinical manifestation, the patient was qualified for conservative treatment under control of the orthopedist and the neurosurgeon.

## 3. Discussion

The occipital condyle fractures are rare injuries, and their incidence is estimated at 0.4%–0.7% of all adult patients suffering from severe injury [1]. The number of the occipital condyle fractures in children is even lower [2]. The most common cause of the occipital condyle fractures is traffic accidents [2, 3]. Fractures of the occipital bone are rarely diagnosed based on classic plain X-ray images. For this reason, the diagnostics should be extended with CT-scan of the head with the visualization of the superior part of the cervical segment of the spine in all patients with suspected injury of the craniocervical junction reporting pain symptoms even if plain X-ray images seem to be normal. CT-scan is the best diagnostic tool for instant confirmation of the diagnosis [4–6]. Early diagnosis and suitable treatment are critical in these cases. The literature provides many contradictory reports regarding use of MRI as the diagnostic tool in the occipital condyle fractures [4, 5, 7, 8]. MRI constitutes supplement to CT-scan, because it allows evaluating damage in the ligamentous apparatus of the craniocervical junction and potential damage of the medulla oblongata/the spinal cord at this level. MRI is also source of information in case of accompanying neurological deficits, especially at the side of the cranial nerves and in case of suspicion of accompanying vascular damage. There are two commonly used classification systems for aforementioned fractures, according to Anderson-Montesano and according to Tuli et al. [1, 3–5, 9]. Anderson-Montesano classification defines three types of fractures. Type I includes comminuted fracture of the condyle resulting from mechanism of axial compression and type II includes basilar skull fracture extending to the occipital condyle. Both types of fractures are usually stable in nature. Type III includes avulsion type of fractures occurring as a result of disruption of various size of bone fragment of the condyle near the alar ligament, and this type of fracture is usually instable in nature. Tuli's classification differentiates two types of fractures. Type I includes undisplaced and stable fractures. Type II includes two subtypes. Type IIA includes displaced and stable fractures. Type IIB includes displaced fractures with instability at the level of occiput-C1/C2. There is no consensus in terms of superiority of one classification system over the other one. Classification of fracture type frequently created problems, especially differentiating of type I with type III stable fracture according to A-M [7]. In 2012, the classification system was published, which classified the occipital condyle fractures into unilateral or bilateral and the ones with or without accompanying atlantooccipital displacement [8]. The injuries accompanying the occipital condyle fractures described in the literature as occurring in children include the following: intracerebral injury and injury of the spinal cord and their consequences in terms of paresis/paralysis of the limbs and neurological deficits in inferior cranial nerves (IX–XII, most commonly, including nerve XII, which is related to close proximity of the occipital condyle with the hypoglossal canal) [2]. It is worth emphasizing that none of available publications established presence of delayed deficit in cranial nerves in children, which are consequence of secondary displacements or forming hypertrophic osseous callus [10]. Injuries of the spinal cord accompanying the occipital condyle fractures are more commonly described in children than they are in adults. In available literature, we also did not find any description of posttraumatic extradural meningeal spinal cyst accompanying the occipital condyle fracture. In addition, the case of our patient is the only described case of posttraumatic extradural meningeal spinal cyst located at the ventral side of the spinal cord within the cervical segment of the spine. Taking into account the level and location of the extradural meningeal spinal cyst in our opinion there is no direct connection between the occipital condyle fracture and development of the cysts. Cyst formation was most likely the result of spinal injury at a lower level. The meningeal spinal cysts are very rare constituting only 1% of all tumors of the spinal cord [11]. They may occur at any level of the spinal cord, but, most commonly, they are located in the central or inferior segment of the thoracic spine [12, 13]. MRI of the spine is the best diagnostic tool to diagnose the spinal cyst. These lesions are the best visible in the sagittal sections of the spine in T1-weighted images [14, 15]. CT myelogram is the best evaluation method, which allows detecting defect of the dura mater constituting the cyst pedicle, which is the place of communication of the cyst with subarachnoid space [16, 17]. The literature also describes diagnostic method, which provides evaluation of pulsating flow of cerebrospinal fluid within the cyst pedicle with use of kinematic MRI [17, 18]. Kinematic MRI also allows evaluating mechanism of the spinal cord compression by the mass of the cyst [18]. Meningeal cysts were classified by Nabors et al. into three types. Type I includes extradural meningeal cyst without neural tissue. Type II includes extradural meningeal cyst containing neural tissue. Type III includes intradural spinal arachnoid cyst. In addition, type I is divided into two subgroups: type IA, extradural spinal arachnoid cyst, and type IB, sacral meningocele. Type IA meningeal cysts occur as a result of protrusion of the arachnoid due to congenital or acquired meningeal defect. The most common location of this type of cysts is dorsal part of the central and inferior segment of the thoracic spine. Pathogenesis of occurrence of type IB of the cysts is not entirely known, and, most likely, they are congenital in nature. Type II of the cysts includes the periradicular cyst and Tarlov cyst. Type III of the cysts most commonly occurs as a result of the inflammatory process of the arachnoid caused by injury, hemorrhage, or inflammatory process. In 2009, the authors of the publication [18] proposed modification of classification developed by Nabors et al. dividing type I of the cysts into the ones which have connection with the subarachnoid space (type IA) and the cysts without connection with the subarachnoid space (type IB). Children with diagnosed extradural meningeal spinal cyst may demonstrate various clinical manifestations depending on location and size of the lesion. These symptoms are caused by compression of the spinal cord or the nerve roots by the cyst, and the most common are the following: back pain, radicular pain, sensory disturbances, muscle weakness, muscle atrophy, gait abnormalities, paresis/paralysis of the limbs, urination disturbance, and defecation disturbance [13, 19]. A few mechanisms have been described, which are responsible for enlarging the extradural meningeal spinal cysts: (1) “valve” mechanism forcing one-way flow of the cerebrospinal fluid into the lumen of the cyst, (2) hyperosmotic content of the cyst forcing water diffusion through the wall of the cyst, and (3) active secretion of the fluid by the arachnoid cells of the cyst wall [18]. Unsteady nature of symptoms with periodical remissions and relapses occurs in ca. 30% of the patients and it is most likely caused by temporary increase in pressure of the cerebrospinal fluid as a result of change in body position and Valsalva mechanism [16, 20]. Majority of the authors recommend conservative treatment in case of the cysts with small size and the ones with asymptomatic course. However, such cases require strict neurological/neurosurgical and imaging control. Surgical treatment is indicated in patients with symptomatic cysts [19, 21]. Surgical excision or fenestration/drainage are treatment methods of choice. Fenestration or drainage is indicated in case of lesions located in front of the spinal cord [22]. Selective closure of the cyst pedicle based on images obtained from CT myelography or kinematic MRI was also described as the therapeutic method [17]. It is a consensus that efficacy of treatment is ensured by total surgical removal of the lesion and repair of the dura mater defect [13, 19, 22].

## 4. Conclusions

We have presented the case of a girl, who suffered from the avulsion fracture of the right occipital condyle (type III according to Anderson-Montesano classification) complicated with the posttraumatic extradural meningeal spinal cyst located at the ventral side of the spinal cord in the cervical segment of the spine. Presented case proves that diagnostics of the occipital condyle fracture should be extended with MRI of the head and the cervical segment of the spine not only to evaluate damage of the ligamentous structures at the level of C0-C1-C2 but also to monitor healing process and detection of potential complications. This finding in our opinion has major clinical significance.

1. The best method for diagnosing the occipital condyle fracture is CT-scan evaluation of the craniocervical junction.
2. Defining morphology and stability of the fracture in CT/MRI evaluation but not assigned type of fracture is crucial in selection of a suitable treatment method.
3. MRI of the head and the cervical segment of the spine is a good tool for monitoring process of healing of the ligamentous damage of the craniocervical junction and detection of potential complications.
4. Extradural meningeal spinal cysts are rare complication of the spine injuries.
5. Surgical treatment of the extradural meningeal spinal cysts is indicated in patients with accompanying symptoms of the spinal cord or the nerve root compression.
6. Kinematic MRI is the future of diagnostics and deciding on method of surgical treatment of the extradural meningeal spinal cysts.
